# Service user and family participation in mental health policy making in Timor-Leste: a qualitative study with multiple stakeholders

**DOI:** 10.1186/s12888-020-02521-3

**Published:** 2020-03-12

**Authors:** Teresa Hall, Ritsuko Kakuma, Lisa Palmer, Harry Minas, João Martins, Greg Armstrong

**Affiliations:** 1 Nossal Institute for Global Health, The University of Melbourne, Exhibition Street, Melbourne, 3004 Australia; 2 London School of Hygiene and Tropical Medicine, London, UK; 3 Centre for Mental Health, The University of Melbourne, Melbourne, Australia; 4 School of Geography, The University of Melbourne, Melbourne, Australia; 5 Faculty of Medicine and Health Sciences, National University of Timor-Leste, Dili, Timor-Leste; 6 Nossal Institute for Global Health, The University of Melbourne, Melbourne, Australia

**Keywords:** Mental health policy, Participation, Participatory governance, Timor-Leste, Asia-Pacific

## Abstract

**Background:**

Participation in mental health system strengthening by people with mental health problems and their families is a cornerstone of people-centred mental health care, yet there is a dearth of research about participation from low- and middle-income countries (LMICs), particularly from the Asia Pacific region. Hence, this study aimed to assess the current situation, challenges, enabling factors and future actions for service user and family participation in mental health policy making in Timor-Leste.

**Methods:**

In-depth interviews were conducted with 85 adults (≥18 years) who were: (1) mental health service users (*n* = 20) and their families (*n* = 10); (2) government decision makers (*n* = 10); (3) mental health and social service providers (*n* = 23); (4) civil society (*n* = 9); and (5) other groups (*n* = 13). Interview data was analysed using framework analysis.

**Results:**

There was limited service user, family and community participation in mental health policy making in Timor-Leste. Perceptions that policy making is a technical exercise and that people with mental health problems lack cognitive capacity, and a lack of supportive mechanisms challenged participation. Enabling factors were a strong focus on human rights within the social sector, and existing mechanisms for advocacy and representation of people with disabilities in social policy making. Participants suggested bolstering civil society representation of people with mental health problems, and increasing mental health awareness and literacy, including government competencies to facilitate service user participation.

**Conclusion:**

The findings highlight the need for theoretical and practical focus on the role of family within mental health system development in LMICs. Global mental health research and practice should adopt a critical approach to mental health service user and family participation to ensure that the concept and strategies to achieve this are embedded in LMIC knowledge.

## Background

Participation in mental health system strengthening by people with mental health problems and their families is a cornerstone of people-centred mental health care [1, 2]. Participation is a key strategy of the World Health Organisation Framework on Integrated People-Centred Health Services (WHO IPCHS) [3] and global mental health directives [4, 5]. Participation can occur at micro- (e.g. individual health decision making), meso- (e.g. service delivery and planning) and macro-levels of the mental health system (e.g. governance and policy making) [6]. This article focuses on mental health service user and family participation in public policy making for mental health.

Service user participation in policy making reflects a broader shift to increase citizen involvement in governance through new public management [7, 8]. From this perspective, participation has instrumental value in that beneficiaries’ knowledge is used to maximise health system effectiveness [7, 8]. Participation is also driven by human and disability rights movements, including those for psychiatric survivors and people with psychosocial disabilities [9, 10]. Participation is a core principle in the United Nations Convention on the Rights of Persons with Disabilities (UNCRPD) [11]. From this perspective, the involvement of people with mental health problems in policy making serves to combat unsupportive social and systemic barriers to their full participation in society [12].

Participation of mental health service users and families in health governance is difficult to achieve. Across all countries, community-member participation is often limited to health promotion rather than involvement in higher-level health decision making (e.g. participation in implementing a health intervention rather than defining the health problem to be targeted, designing the intervention or participating in key project governance structures) [13]. People with mental health problems face additional and unique barriers to participation. A large body of research predominantly from high-income countries (HICs) has identified attitudinal (e.G. *stigma*), logistical (e.g. financial and time costs) and structural barriers (e.g. lack of participatory mechanisms) to participation in policy making by people with mental health problems [1, 14–19].

Mental health civil society organisations are mechanisms that increase the agency of people with mental health problems to make decisions about their own lives, including participation in governance processes [1, 20, 21]. Organisations for mental health service users, people with psychosocial disabilities, and survivors of psychiatry have emerged across HICs and low- and middle-income countries (LMICs) with different mental health service histories (i.e. psychiatric institutionalisation, community-based mental health care) [10, 22, 23]. Mental health civil society organisations are still developing throughout the Asia and Pacific region [10], and there is a dearth of evidence about the challenges and opportunities for mental health service user and family participation in LMICs in this region. This study aimed to fill this knowledge gap by investigating stakeholder perspectives on mental health service user and family participation in mental health system strengthening in Timor-Leste, a lower-middle income country in South-East Asia.

Timor-Leste is a small island nation of 1.3 million people in the process of strengthening its mental health system [24]. There are no rigorous, up-to-date population estimates of mental distress in Timor-Leste, but the 2016 Global Burden of Disease study estimates a 11.6% prevalence of mental and substance use disorders [25]. Population mental health in Timor-Leste is influenced by known risk factors of mental distress, including high rates of poverty, unemployment, and past and present experiences of violence and trauma [26–28]. During Indonesia’s occupation of Timor-Leste from 1975 to 1999, between 102,800 and 183,000 people are estimated to have died, including 18,600 people from unlawful killing or being ‘disappeared’ [29].

Health system strengthening has been a primary development target over the two decades since Timor-Leste’s independence from Indonesia [30, 31] and has included the development of some mental health system infrastructure. Government mental health care is predominantly community-based and integrated into primary health care [32]. Non-government organisations (NGOs) provide a psychosocial rehabilitation service (Pradet), long-term stay service (Klibur Domin) and inpatient psychiatric service (São João de Deus, Laclubar). There are also social sector and civil society efforts to promote and protect the rights of people with disabilities, including people with mental health problems [33].

The yet-to-be implemented Timor-Leste National Mental Health Strategy 2018–2022 prioritises participation by people with mental health problems and their families [26]. While Timor-Leste has disabled persons organisations (DPOs) to support and advocate for those with disabilities, there is no mental health service user or family organisation, or research in Timor-Leste to support service user and family participation in mental health governance. Hence, this study aimed to assess perspectives about the current situation, challenges, enabling factors and future actions for participation of mental health service users and their families in mental health policy making in Timor-Leste. This research builds upon previous research by the authors that was conducted to inform development of Timor-Leste’s National Mental Health Strategy [34].

## Methods

### Setting

The cross-sectional qualitative study was conducted across multiple levels of the mental health system in Timor-Leste. Dili, the administrative and political capital of Timor-Leste, was selected because it is the site of national level policy making and the base of most government and NGO mental health service providers. Baucau municipality, and its administrative post, Venilale were selected to understand health system governance at the local level. Baucau has the largest sub national government administration in Eastern Timor-Leste and a population of 123, 203 persons [35]. The population characteristics of Baucau and Venilale align with Timor-Leste’s national average. Each have: a median age of 19 years, approximately 5.5 persons per average household, employment rates around 50, and 1.2% population proportion of mental illness, although the latter is likely to be underestimated [35]. Laclubar administrative post in Manatuto municipality was also included because it hosts the São João de Deus inpatient mental health facility, which was the only such facility in Timor-Leste at the time of the study.

### Participants

The study sought to understand the perspectives of multiple stakeholders about mental health service user and family participation given the early stage of mental health system development in Timor-Leste. In-depth interviews were conducted with 85 adults (≥18 years) who were: (1) mental health service users (*n* = 20) and their families (*n* = 10); (2) government decision makers (*n* = 10); (3) mental health and social service providers (*n* = 23); (4) civil society (*n* = 9); and (5) other groups including international development organisations (*n* = 13, see Table 1). Mental health service users and their families were identified and invited to participate in the study by administrative post health staff in Venilale and Baucau, and NGO service providers in Dili. Author TH met mental health service providers at each location to explain the purpose and requirements of the study and made clear the need for potential participants to have capacity to give informed consent to participate in the interview. Service providers recruited mental health service users and their family members in-person or on the phone. Notification of potential participants by trusted sources was important in Timor-Leste because of the strong role of familial or kin connections in determining perceived trust and safety. Subsequently, Author TH and the interpreter met potential participants, explained the purpose and requirements of the study Tetum and invited them to participate in the study. Inclusion criteria were: 18 years or older; current or previous use of health or social services for their/family members’ mental health problems; and ability to provide informed consent and respond to interview questions. In the absence of a Timorese culturally validated psychiatric diagnostic tool, the definition of mental health service user was intentionally kept broad to capture the range of people who were considered to have mental illness by trained service providers. Government decision makers, service providers, civil society and other groups (participants groups 2 to 5) were purposively recruited by First Author TH based on their job responsibilities. Data were collected from September 2017 to August 2018.

**Table 1:** Participant demographics

	Mental health service users (MHSU)		Family members (FM)		Service providers (SP)		Decision makers (DM)		Civil society(CS)		Other community members and organisations (OT)		Total	
N	20		10		23		10		9		13		85	
	n	%	n	%	n	%	n	%	n	%	n	%	N	%
Age														
26–40	12	60	2	20	10	43.5	1	10	4	44.4	6	46.2	35	41.2
41–55	6	30	5	50	8	34.8	8	80	3	33.3	5	38.5	35	41.2
56–70	2	10	3	30	5	21.7	1	10	2	22.2	2	15.4	15	17.6
Gender														
male	7	35	7	70	13	56.5	9	90	8	88.9	7	53.8	51	60.0
female	13	65	3	30	10	43.5	1	10	1	11.1	6	46.2	34	40.0
Education														
none	1	5	2	20	0	0.0	0	0	0	0.0	0	0.0	3	3.5
primary	11	55	5	50	0	0.0	0	0	0	0.0	0	0.0	16	18.8
secondary	4	20	1	10	1	4.3	0	0	4	44.4	3	23.1	13	15.3
tertiary	4	20	2	20	22	95.7	10	100	5	55.6	10	76.9	53	62.4
Location														
Dili	5	25	0	0	15	65.2	5	50	6	66.7	9	69.2	40	47.1
Baucau	2	10	1	10	4	17.4	4	40	0	0.0	3	23.1	14	16.5
Venilale	13	65	9	90	3	13.0	1	10	3	33.3	1	7.7	30	35.3
Laclubar	0	0	0	0	1	4.3	0	0	0	0.0	0	0.0	1	1.2

Table adapted from [36]

### Data collection

Interviews were semi-structured using an interview guide tailored to participant type. The full interview guide was from a broader study investigating people-centred mental health care in Timor-Leste (see full interview guides in Additional file 1). Interview questions pertaining to the current study enquired about the experiences of and roles for service users and their families in the mental health system. Participants were asked: “*When the government of Timor-Leste makes decisions about what services are needed to help people who have mental health problems, are mental health service users and their families included in these decisions? Should they be? What makes this difficult? What would help this to happen*?” The interview guide was translated, and its meaning checked and piloted before data collection commenced. Author TH conducted all interviews directly in English (*n* = 25) or with a trained interpreter in the national languages Tetum (*n* = 48) or Portuguese (*n* = 1), or one of several Baucau local languages (Makassai: *n* = 7, Cairui: *n* = 4). Interviews lasted on average 47 min (range: 7 to 111 min), and were in private places, including workplaces, health facilities and community houses. Recruitment of interview participants was discontinued when data saturation was reached; i.e. when subsequent interviews revealed no new information [37].

### Data analysis

Framework analysis, an inductive and deductive qualitative data analysis method [38], was used to analyse interview data in NVivo version 12 [39]. Framework analysis was suitable for this applied study because the technique is not aligned with any specific epistemological stance and places the research questions at the forefront of the analysis [38]. Framework analysis consisted of the following seven stages specified by Gale (2013) [38]: transcription, familiarisation with the data, coding of a priori and emergent themes, development of a working analytical framework, applying the analytical framework, charting into a framework matrix and interpreting the data. Author TH transcribed the interviews, read and re-read all transcripts before coding five transcripts using a combination of emergent themes and a priori themes based on the overall project research questions. The a priori themes relevant to the current study were: (1) current situation, (2) enabling factors, (3) challenges and (4) suggested future actions for participation of mental health service users and families in policy making. A second researcher on the project reviewed and commented on the working analytical framework with reference to the five interview transcripts. The two researchers discussed the framework and agreed to make minor changes such that codes were based on larger thematic categories to most efficiently deal with the large volume of qualitative data generated from the 85 people interviewed. Author TH then applied the refined analytical framework to all transcripts. This article reports four main themes and 11 sub-themes relevant to mental health service user and family participation. Preliminary results were presented back to participants and interested parties in communities in Dili and Venilale to verify the authors’ interpretation of the data.

### Ethics

Verbal or written consent (depending on participant preference and literacy) was provided before interviews commenced and were audio recorded. Ethical approval was granted by University of Melbourne Human Ethics Sub-Committee (HESC: 1749926) and National Institute of Health in Timor-Leste (1070MS-INS/DE-DP/CDC-DEP/IX/2017).

## Results

The results section is structured around the study aims to investigate the following aspects of mental health service user and family participation in policy making in Timor-Leste: (1) current situation, (2) challenges, (3) enabling factors, and (4) suggested future actions (see Table 2).

**Table 2:** Themes and sub-themes for mental health service user and family participation

Theme	Sub-themes
Current situation	Experience of participation/non-participation in policy
	Experience of participation/non-participation in advocacy
Challenges	Novel idea
	Perceived incapacity of people with mental health problems
	Persistent stigma
	Perceived need for technical expertise
Enabling factors	Human rights discourse
	Disability rights and existing structures
	Lived experience as expertise
Future actions	Mental health service user forum, network or organisation
	Knowledge and awareness

### Experience of participation

None of the mental health service users interviewed had personally participated in policy making. However, decision makers, service providers and civil society members reported that some people with mental health problems and their families participated in the development of the National Mental Health Strategy 2018–2022 alongside inter-ministerial, NGO, civil society and community representatives. One service provider described working with families affected by mental ill-health during consultation for this Strategy in Venilale:

*It was really good because all the families came, all the patients came for the duration of the day. We ate together, drank [coffee] together and made decisions together, made the strategic plan together* (SP005, 56-60 years, male).

Mental health service users reported participating in several mental health advocacy events in Dili and Venilale, including the annual World Mental Health Day and World Disability Day celebrations. Although these advocacy events were not policy making, service users considered these events to be important occasions for inclusion. One female service user from Venilale relished the opportunity to attend such public events:

*I like to go to activities run by the health centre. Normally [the community] go to activities hosted by the administrative leader, [the leader] invites us [me and my family] to come* (P007, 36-40 years, female).

Another service user explained that he valued performing in an opening dance at the World Disability Day celebrations in Dili:

*I like to dance because when I dance it reminds me of when I was little, before I was sick, and I was involved in dancing with my community* (P016, 36-40 years, male).

### Challenges

For many participants, including mental health service users and their families, it was a novel idea that people with mental health problems would be involved in health policy making. Many mental health service users reported that they spent most of their time in and around their homes: *“normally, every day I stay at home. When it starts to get hot in the house, I’ll move and sit here [under a tree in the shade]*” (P009, 66–70 years, male). As a result, there was a large disconnection between the daily lives of people with mental health problems and the policy making arena, particularly for those who lived in rural areas away from administrative institutions. Participants from other groups were also confused by the suggestion of mental health service user participation in policy making. When asked whether mental health service users had been involved with government decision making, one community member from Venilale responded:

*Not yet, they haven’t. But one time I asked the Ministry of Social Solidarity to give rice to the patients* (OT005, 66-60 years, male).

Participants across groups explained that national policies should be made by government officials who possessed the requisite technical expertise, not community members. One [participant] stated:

*[Policy making is] a technical thing, that [government officials] understand, have the knowledge, and are the ones that need to make the decisions. And there is this understanding that there has to be dissemination at the community level, so [government officials] go and pass the message of the decision that the government has already taken.* (OT009, 46–50 years, female)

Part of this technical expertise was related to education. One decision maker explained that people with mental health problems may be able to contribute if they were educated, but otherwise could not:

*It depends on the education of the crazy person. If he doesn’t really have enough knowledge then he doesn’t know what to do for the government*. (DM001, 51–55 years, male)

Stigmatising beliefs that mental health service users lacked cognitive capacity were a major challenge to their participation in health policy development. Multiple decision makers, service providers and some disability civil society members expressed this idea:

*… because policy discussions require thinking* (points at head), *and people with mental health problems cannot discuss* (crosses temple) (DM003, 46-50 years, male).

One male family member couched this lack of capacity in terms of needing to facilitate his wife’s contact with the mental health system:

*Being in a family, it is very important that when a husband or a wife has problems with mental illness, their partner supports them. If the medications are not at the health centre, I find it hard to find the medications to give to my wife. These are part of how I support her* (FM002, 61-65 years, male).

One disability organisation member stated that the population in general: “*don’t respect what people with disabilities are thinking*” (CS003, 26–30 years, female). One social service provider said that she could not trust people with mental health problems to consistently have the capacity to contribute to health policy development:

(sighs) *I don’t know, because sometimes they can talk to us as if they don’t have a mental illness, but other times, everything has changed, and they can't remember us anymore* (SP015, 31–35 years, female)

This contrasted with accounts from some mental health service users that they were able to ‘think’ even when they were unwell:

*“Even when I went to Dili [for treatment when I was unwell], my brain was still normal. I could still decide my own objectives and where to go and find a solution. It was just that I felt afraid, felt scared*.” (P012, 26–30 years, female)

Some civil society participants agreed that people who had recovered could participate, however other decision makers and service providers thought that the broader community may still not trust the judgment of people with mental health problems:

*Timor is very small, people know each other. If [someone] becomes crazy, even if they become well again, people understand that they have this background of mental illness. The communities understand that sometimes they might become unwell again, so it would be a problem for them to participate.* (DM001, 51–55 years, male)

### Enabling factors

Within the social sector, a strong focus on human rights emerged in the interviews that aligned with the concept of mental health service user and family participation. Multiple participants across groups employed human rights language and cited human rights training they had received from disability organisations. One Ministry of Health representative explained that the ministry began to focus on the social inclusion of people with mental illness: “*when we considered that it is a human rights problem, so we have to treat everyone the same*” (DM001, 51–55 years, male). A service provider from Venilale referred to common humanity as a reason for improving the lives of people with mental health problems: “*People with mental illness are humans like all of us, so they have to enjoy their life*.” (SP002, 41–45 years, female).

Social sector participants reported existing structures for representation of people with disabilities in social policy making. The National Disability Strategy and accompanying Action Plan were upheld by several disability sector participants as examples of participation by multiple stakeholders:

*[The National Disability Strategy received] really good input from service providers, people with disabilities themselves and their families, and also the UN, agencies, donors* (OT002, 31-35 years, female).

A Ministry of Social Solidarity and Inclusion representative confirmed that some people with psychosocial disability were included in this consultation by creating a safe space:

*When [people with psychosocial disability] came and gathered with us, we did not see them as a different human being or different from us; nobody said 'that person is crazy', it didn't happen. But I don't know if that happens outside the meeting, in [the person's] house or community, if people are bullying him.* (DM009, 46–50 years, male)

Some service providers and civil society participants also described the value of including people with mental health problems in health policy development so they could share their experiences with policy makers. One civil society participant described lived experience of mental illness as a type of expertise in itself:

*The government need to include them so that they can present themselves. To say ‘I suffered from mental health problems but I received treatment, and now I am back to being a normal person. Now I am in front of you and I would like to contribute my ideas.’* (CS009, 61–65 years, male)

### Future actions

Several participants across groups suggested ways to increase participation by people with mental health problems and families in health policy making. They first identified a need to raise awareness about mental health to increase participation of people with mental health problems more generally in society. One male family member described how his unwell brother-in-law had received a better reception from the community after community members understood more about his condition.

*The community understand him because he has been sick for a long time. [Before] sometimes they came and called him bad words, but then I tell them not to say bad words to him. So all the people here know and understand [our situation]. Sometimes he goes and takes other people’s belongings, and people understand. But sometimes the neighbourhood kids come and annoy him, and I am the one that tells the kids not to do this.* (F015, 46–50 years, male)

Multiple participants believed that decision makers from the community to national levels needed increased knowledge and support to facilitate the involvement of people with mental health problems in policy making. One decision maker explained his concerns:

*We [the government] would need to have knowledge about how to do this because this is not a normal process. [Based on] if the person is thinking clearly, if they are well or not. We have to think deeply to make political decisions, [so it might be complicated] if we have to bring [people with mental illness] all together to have a discussion.* (DM003, 46–50 years, male)

One participant described how local governance structures could be used to support participation by families affected by mental illness across *all* policies:

*[Participation could be promoted] through the community base, through the town chiefs and others to create more awareness [of participation] and to involve them [people with mental health problems]. So that there is leadership at the lowest level, at the village level, from people that [people with mental health problems] feel comfortable approaching. But this is true for all policies that affect them, not just the mental health policy*. (OT013, 46–50 years, male)

Several service providers, decision makers and civil society participants said that participation could be facilitated through the establishment of a forum, network or organisation led by people with mental health problems. One disability sector representative explained:

*I think a forum would provide that safe space for [people with psychosocial disabilities] to talk about their experiences, among themselves to start with, and then create that confidence to talk about it [more broadly].* (OT002, 31–35 years, female)

This was reflected by one mental health service user who said that she appreciated gathering with fellow mental health service users at NGO, Pradet:

*I’m really happy and I like it at Pradet. Before I came to Pradet, I couldn’t remember or talk about anything. But coming to Pradet makes me happy and I start to remember things and talk about things*. (P018, 31–35 years, female)

A service provider also recognised that a mental health service user organisation could encourage individuals with mental illness to use their voices:

*[There is one mental health service user] who’s at university, yeah but he’s pretty shy and retiring. So while nobody stands out there [advocating], there are a number of fairly good individual stories.* (SP009, 66–70 years, female)

Another service provider discussed how such a forum might enable future participation:

*I wonder whether you'd need a precursor [to participation...] what may need to come first would be the support groups, the advocacy groups, people who have recovered from mental illness or have a persisting mental illness that they manage [who] might be able to [...] advocate for better rights for mental health patients, better access to services.* (SP013, 36–40 years, male)

However, the same disability sector representative quoted above said that civil society representation of people with mental health problems was challenged by a lack of resources to bring mental health service users from all over Timor-Leste together to participate. She also spoke about the potential challenge of gatekeeping by families:

*So I think the challenge is getting the clients [to come together], and their families to want to allow them to be part of this.* (OT002, 31–35 years, female)

## Discussion

This study is the first to investigate the current situation, challenges, enabling factors and future actions for mental health service user and family participation in mental health governance in Timor-Leste. The key findings were:

1. There was limited participation in mental health policy making and advocacy events by people with mental health problems, families and communities in Timor-Leste.
2. Challenges identified to participation were perceptions that policy making is a technical exercise and that people with mental health problems lack cognitive capacity; and a lack of supportive mechanisms to facilitate participation.
3. Enabling factors were a strong focus on human rights within the social sector, and existing mechanisms for advocacy and representation of people with disabilities in social policy making.
4. Participants suggested bolstering civil society representation of people with mental health problems, and increasing mental health awareness and literacy, including government competencies to facilitate service user participation.

The limited participation of people with mental health problems, their families and communities in mental health policy making in Timor-Leste, and its interpretation as a novel idea have been reported in other LMICs, including Nepal, Ethiopia, India and Nigeria [16, 19, 21, 40, 41]. Participation in policy making by Timorese people with mental health problems and their families was beset by the practical and social challenges it sought to address [42]. Practically, there was no service user organisation to facilitate individual and collective engagement of Timorese people with mental illness, who have limited opportunities to develop advocacy skills due to their exclusion from society and public services (i.e. education, social welfare, legal) [36]. Socially, Timorese norms do not legitimise the role of people with mental health problems in decision making [43], particularly for high level national policies, as has been found in other LMICs [16, 19]. Hence, Timorese people with mental health problems can be seen to experience the two forms of what Fricker coins ‘epistemic injustice’: (1) testimonial injustice such that their voices are not taken seriously without corroboration from another source, and (2) hermeneutical injustice such that there is no collective framework through which to understand their experiences [44].

Strengthening civil society representation of Timorese people affected by mental ill-health seems necessary to facilitate their participation in mental health system development. However, unlike HICs and some LMICs in which people have mobilised around common experiences of mental health service use [20, 22, 23], Timorese people with mental health problems may not identify as ‘service users’ because of the limited availability of mental health services in the country [32, 43]. As a result of barriers to mental health care access, the service user population may also be constituted by people with longer-term or complicated experiences of mental illness, who are known to be less likely to participate in HICs [14, 15]. The heterogeneity of Timorese sociocultural explanatory models of mental ill-health (e.g. spiritual or ancestral imbalance, natural, biological, environmental causes) [34] may also disguise any shared realities among people experiencing mental distress.

Instead of a health framing, Timorese people with mental health problems may mobilise to promote a disability rights narrative. This latter frame may focus on reducing the community-based and systemic exclusion confronted by Timorese people with psychosocial disabilities (e.g. exclusion from education, employment, confinement and physical restraint) [36] and the combating of the biomedical model of mental illness, which is a well-documented challenge to social participation for people with mental health problems [45–47]. Disability rights have been a powerful impetus for uniting people with psychosocial disabilities in some countries in the Asia Pacific [10], and there is already a strong disability sector in Timor-Leste for people with physical and sensory disabilities [48]. However, Timorese people with psychosocial disability are not currently active in the disability sector, and members of the disabled community have been found to hold stigmatising attitudes about people with mental health problems [43]. This suggests that an enabling environment for mental health service user and family participation in mental health policy development in Timor-Leste may be a staged process. In the first instance, ratification of UNCRPD and training is needed for decision makers and disability organisations to meaningfully include people with mental health problems [16, 19]. Specifically, decision makers and front-line service providers of social, legal, violence support and educational services, among others, should be equipped to know how to make reasonable accommodations for people with mental health problems within their standard intake, assessment and service procedures, and promote inclusion through the service process (e.g. by having a trusted person present if desired, fostering a safe space, providing a range of ways to communicate information).

An important but neglected element of civil society mobilization around mental health in LMICs also concerns family involvement in these networks. The family movement was a key impetus for increased focus on participatory mental health governance in Western HICs [49]. In many HICs, mental health service user and family participation are now approached separately to account for the unique, and sometimes conflicting, needs and views of these identity groups [15, 50] [51]. However, previous research in Timor-Leste identified a central role of family in mental health such that individual needs and preferences are defined and enacted in relation to family [36]. Hence, further consideration is needed to ensure that participatory mechanisms for mental health reflect Timorese sociocultural organisation around family.

On the other hand, given Timor-Leste’s broader development challenges, participation in health policy may not be a priority for many Timorese families affected by mental illness when they are preoccupied with more urgent practical challenges (e.g. poverty, financial and food security, experiences of violence) [28, 52, 53]. Research from India and Nepal found that the focus on human rights advocacy by national-level (elite) disability activists did not reflect the concern for basic needs expressed by people with disabilities at subnational levels [54]. Interest in participation is also likely to be influenced by service use. Abimbola found that Nigerian communities were less likely to mobilise around issues of primary health care governance when they had the option of seeking alternative care [55, 56]. Given the reliance on and preference for customary healing in Timor-Leste [34, 57], involvement in formal policy making may not seem relevant to many Timorese people. This underscores the importance of facilitating participation of families affected by mental illness in existing local governance structures. It also highlights the need to ensure that future mental health advocates accurately represent the diverse, localised concerns of people with mental illness in Timor-Leste.

This study had several limitations. In-depth interviews collected comprehensive accounts of participation from multiple perspectives, however the findings cannot be generalised. Mental health service user and family participants from Venilale, Baucau and Dili may not represent the views of these groups in other parts of Timor-Leste, or people who do not engage with mental health services. These participants also did not have direct experience in policy making, which limited the scope of the findings. However, given that there is minimal service user and family participation in mental health policy in Timor-Leste, the views of service user participants may reflect those of the general service user population more so than people who had previously participated.

Future research could investigate the burgeoning civil society representation of people affected by mental health problems in Timor-Leste, with careful attention to the role of family in these emerging networks. Participatory action research methodologies could also be employed to encourage mental health service users and their families to define participation on their own terms as well as building their skills and capacities for participation through the research process [58].

## Conclusion

This study has made an important contribution to understanding mental health service user and family participation in policy development in Timor-Leste. The findings also provide insights about participatory mental health governance in the Asia and Pacific region, where there is a dearth of research. Although the study identified many of the same barriers to mental health service user and family participation reported globally, it highlighted the need for theoretical and practical focus on the role of family within mental health system development. While the human rights discourse was identified as an enabling force for participation in Timor-Leste, its individualistic formulation has been criticised for ignoring familial and relational factors that shape life and health in many LMICs [59–61]. The study findings also challenge the positioning of participation as a panacea to mental health system strengthening in LMICs when people’s priorities are informed by broader and more urgent development concerns. Global mental health research and practice should adopt a critical approach to mental health service user and family participation to ensure that the concept and strategies to achieve this are embedded in LMIC knowledge.

## Supplementary information

**Additional file 1.** Interview Guides. The full interview guides for participant stakeholder type in English and Tetum language.

## Data Availability

Participants shared their opinions and experiences upon assurance that their confidentiality and anonymity would be protected. Hence, the research data is not available publicly because this would compromise individual privacy and our ethical approval conditions.

## Abbreviations

CS: Civil society member
DM: Decision maker
DPOs: Disabled persons organisations
FM: Family member
HIC: High-income country
LMIC: Low- and middle-income country
MHSU: Mental health service users
NGO: Non-government organisation
OT: Other community member
SP: Service provider
UNCRPD: United Nations Convention on the Rights for Persons with Disabilities
WHO IPCHS: WHO Framework on Integrated People-Centred Health Services
WHO: World Health Organisation
